# The Lived Experience of Older Adults with Monitoring Technologies: An Interpretive Phenomenology Study

**DOI:** 10.3390/healthcare14030288

**Published:** 2026-01-23

**Authors:** Alisha Harvey Johnson, Chang-Chun Chen, K. Melinda Fauss, Shu-Fen Wung

**Affiliations:** 1 Sinclair School of Nursing, University of Missouri, Columbia, MO 65212, USA; mmfvfb@missouri.edu; 2 Department of Electrical and Computer Engineering, University of Arizona, Tucson, AZ 85721, USA; changchunchen19@arizona.edu; 3 Betty Irene Moore School of Nursing, University of California, Davis, CA 95817, USA; swung@health.ucdavis.edu

**Keywords:** older adults, monitoring technology, lived experience, phenomenology, aging in place

## Abstract

**Highlights:**

This article reviews an interpretive phenomenological study aimed at understanding the lived experience of older adults with monitoring technologies, specifically on self-identity, independence, autonomy and aging in place. We find that older adults apply a pragmatic approach to assign meaning to a monitored life. This approach reveals perspectives of older adult end users allowing better implementation of monitoring technologies that are acceptable and effective.

**What are the main findings?**

- Older adults use pragmatic strategies to process the meaning of life as “monitored” individuals.
- Self-identity is maintained and technologies are considered useful only if they are tied to meaningful interventions, preferably with a human component.

**What are the implications of the main findings?**

- The inclusion of older adults with dementia is not only possible, but necessary to ensure that all perspectives and end user experiences are understood.
- The insights gained from this research offer valuable insights to ensure that technology-assisted monitoring interventions are effective and acceptable to older adults.

**Abstract:**

**Background:** Most older adults prefer to age in place. Technology-assisted monitoring can enhance safety while maintaining independence. However, there is limited understanding of older adult end users’ preferences and experiences. **Methods:** In this interpretive phenomenological study, we interviewed eight older adults, with and without dementia, to understand their lived experiences with monitoring technology and its impact on self-identity, independence, and aging-in-place. **Results:** We found that older adults use pragmatic strategies to process the meaning of life as “monitored” individuals, reflected in four themes: (1) freedom to age in place, (2) the need for active and integrated intervention, (3) individualized approaches to technology based on temperament, usefulness, and worldview, and (4) a sense of changing situations while remaining unchanged. Adaptive techniques for older adults with dementia successfully elicited complex thoughts and desires when participants were given sufficient time and space. **Conclusions:** As technology-assisted monitoring becomes more common, it is imperative to understand the perspectives of older adult end users. Focusing on lived experiences offers valuable insights to ensure technology-assisted monitoring interventions are effective and accepted as older adults navigate changes in their capabilities and endeavor to age in place.

## 1. Introduction

Most older adults express a strong preference to age in place, signifying a desire for independence. A national survey of older adults in the US found that 77% prefer to age-in-place, allowing them to stay in their own homes while having the option to seek assistance from family and friends, rather than moving into institutional settings [1]. Multiple conditions that can occur as older adults age, such as falls, increasing frailty, and dementia, are significant barriers to aging in a place of the older adults’ choosing. Approximately half of older adults who sustain hip fractures from falls become partially dependent, and 30% become totally dependent on carers, resulting in institutionalization [2]. Older adults with dementia can be institutionalized to address increasing functional and cognitive dysfunction and to relieve the increased strain of caregiving placed on family and friends [3]. Most caregiving is performed by family members, who often lack adequate training in caring for aging loved ones, raising risks and potentially delaying necessary interventions [4,5].

Achieving the goal of aging in place is facilitated by innovations in digital technologies that support the diagnosis, prevention, monitoring, and treatment of chronic medical conditions. Among these domains, continuous monitoring technologies form the most critical foundation for aging in place, as they enable early detection, support prevention, inform diagnosis, and guide timely interventions, while preserving older adults’ independence in their own homes [6,7]. Many modalities have been tested, including remote sensors, wearable technology, the Internet of Things (IoT), and Smart Homes. Among currently implemented monitoring technologies, fall and safety monitoring systems are the most common and most extensively studied applications [8].

Fundamentally, aging in place is not centered on treatment, but on preventing loss of control. Without effective monitoring, prevention becomes generic, diagnosis is delayed, and treatment becomes reactive, ultimately undermining the goal of aging in place [9,10]. The use of technology to monitor older adults can enhance caregivers’ ability to identify increased risks, informing the development of targeted interventions that address unmet needs. Existing research has evaluated these technologies regarding their sensitivity/specificity, usability, and acceptability in older adults [11,12,13,14]. However, little is known about the lived experiences of older adults who are subject to these monitoring devices and must integrate them into their homes, identities, and daily routines. Caring for older adults typically involves collaboration among the older adults themselves, their family carers, and healthcare professionals. It is important to better understand how technology can be useful, valued, interactive, and meaningful for all groups involved. To ensure that technological interventions are acceptable and relevant, we must engage in shared design and consumer-informed design processes [14].

The purpose of this interpretive phenomenological study was to gain a deeper understanding of the lived experience of older adults, both with and without dementia, who are living with monitoring technology. It specifically examined how this technology influenced the older adults’ perspectives on self-identity, independence, autonomy, and aging in place.

## 2. Materials and Methods

### 2.1. Design

Using an interpretive phenomenological (IPA) methodology, we aimed to understand the lived experience of older adults, both with and without dementia, who lived with monitoring technology. IPA was particularly well-suited for this inquiry because it enabled researchers to explore the meaning of experiences by engaging directly with participants. This approach provided detailed insights into how each participant made sense of their experiences, while also allowing researchers to interpret this sense-making through a process known as double-hermeneutics [15]. Ultimately, combining these two perspectives led to a rich and detailed understanding of participants lived experiences with a specific phenomenon—in this case, continuous exposure to monitoring technology. We were specifically interested in how these technologies influenced self-identity, independence, autonomy, and the ability to age in place.

### 2.2. Sample

The study sample consisted of eight residents purposively selected from two long-term care facilities in Missouri and Arizona. These individuals had 9 to 12 months of experience living with fall-monitoring technology, including an ambient radar sensor installed in the ceiling and a pendant-worn accelerometer. Though the monitoring technologies we examined focused on fall-detection, they represented a continuous monitoring presence in the lives of our participants. Therefore, we conceptualized monitoring technology more broadly in this phenomenological approach to encompass all continuous monitoring technologies, not just those related to falls. Participants met the following inclusion criteria: they were ≥65 years old, had experience living with remote monitoring technology for 9 to 12 months, could communicate in English (with or without mobility aids), and could have a diagnosis of dementia or cognitive impairment. In line with purposive, qualitative sampling focused on homogeneous groups, in our case focusing on older adults living with monitoring technologies, sample size was determined by finding saturation (i.e., no new findings were identified in the data) and were in line with typical interpretive phenomenological study size, which can generally range from six to ten [15].

### 2.3. Ethical Considerations

The institutional review board at the University of Missouri approved this multi-site study (IRB# 2107326 MU). Informed consent was obtained from all participants, who were informed of their right to decline to answer any questions that made them uncomfortable and to withdraw from the study at any time. For residents diagnosed with dementia or other cognitive impairments, the PI (MU) or the Co-I (UA) determined the participants’ ability to make informed decisions. Proxy consent was sought from medical power of attorney and/or family members when necessary.

To conduct interviews with older adults with dementia, the PI and Co-PI adjusted their interview timing and techniques to ensure they engaged participants during moments of alertness or lucidity. Interviewers continually monitored participants for their assent in addition to previously obtained consent. If a participant showed signs of distress or chose to discontinue, the interview was paused and rescheduled for a later time when the participant was able to assent and engage in the interview process. In-person interviews were prioritized to allow for ongoing assessment of the participants’ assent and to enhance communication with those living with dementia. Communication techniques were based on the FOCUSED approach, which emphasized essential elements for maintaining calm, directed, and simple communication with individuals with dementia, such as face-to-face interactions, structured dialog, and direct communication [16]. Finally, all data were anonymized, and participants were assigned pseudonyms to ensure privacy and confidentiality.

### 2.4. Data Collection

Data were collected from two long-term care centers providing a range of services from tiered aging-in-place options (based on needs, e.g., assistance with activities of daily living, or meals only) to memory care and assisted living. Prior to the first interview, demographic information was gathered, including age, identified gender, and cognitive status, as measured by Montreal Cognitive Assessment scores (MoCA). Semi-structured interviews were conducted to guide the study aims while also allowing for new lines of inquiry that emerged during conversations, enriching the overall description of the findings. The interviews were audio-recorded for transcription and anonymized during manual transcription verification to ensure confidentiality for data analysis. Field notes were taken and incorporated with participant observations, capturing participants’ responses, interactions, gestures, and facial expressions. This approach helped highlight nuances that might have been overlooked if only audio recordings had been analyzed. Both interview transcripts and fieldnotes were uploaded into qualitative analysis software, Dedoose® version 10 (SocioCultural Research Consultants, LLC, Los Angeles, CA, USA), for inductive analysis. Additionally, reflexive journaling was used throughout the interview process to ensure researchers maintained a hermeneutic approach, focusing on understanding and interpreting participants lived experiences from their perspectives.

### 2.5. Data Analysis

**Figure 1:**
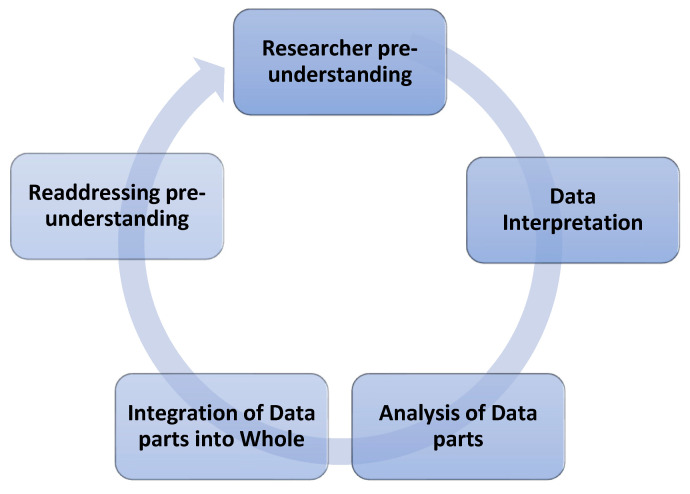
Hermeneutic Circle.

Prior to analysis, all collected data (including transcripts and fieldnotes) were anonymized to protect participant identities. The analysis was conducted using a hermeneutic circle, focusing on a systematic, detailed, and iterative process. Both hermeneutic and IPA methodologies recognized the researcher’s identity as an interpretive tool [17]. The hermeneutic circle enhanced rigor by allowing for “shifting perspectives” of meaning between the parts and the whole, while also acknowledging preconceptions through reflexivity (Figure 1). The PI (AJ) had extensive experience as both a clinical nurse and a researcher in older-adult settings and in the implementation of health monitoring technology. This background provided an insider perspective on the phenomenon under study, which was crucial for sense-making in a double hermeneutic approach [15,17]. The interplay between individual interview segments and the overall conceptualization of findings contributed to confidence in the informed and tested findings [18].

**Figure 2:**
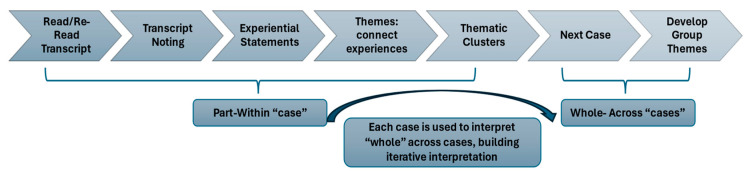
Seven-step IPA process (as adapted from Smith [15]).

An eight-step IPA analysis was conducted, following an adaptation of the process defined by Smith (Figure 2) [15]. Analysis began with familiarization of each transcript and included inductive coding (in Dedoose® version 10) and description of “experiential statements” for each participant (the hermeneutic part). Each participant was initially analyzed to develop preliminary themes within participant cases. Then, each case was compared across participants to develop an overall narrative (the hermeneutic whole), building knowledge and influencing successive interpretations. Iterative reflection allowed the researchers to consider positionality and revisit pre-existing understandings to create and present a cohesive narrative through interpretive methods.

Interpretive processes occurred iteratively at many steps throughout the analysis, propelling knowledge from description to interpretation. For example, experiential statements were created by incorporating PI fieldnotes in an initial effort to integrate participant language use, semantic content and participant meaning with interpretation through the researchers’ knowledge. This interpretive process continued into thematic clustering within individual cases and development into group themes as meaning was interpreted across cases. During the within case analysis, inductive coding and some experiential statements underwent member-checking with select participants to ensure the researchers understood participants’ perspectives and lived experiences. As the analysis progressed into the group-themed, interpretive realm, the analysis was reviewed for consensus by several researchers to increase trustworthiness. Through these intentional steps, the research team was able to incorporate perspectives from multiple adults and researcher perspectives, connecting “parts back to the whole” giving voice to individuals but also finding interconnectedness and actionable findings across the group as a whole [15].

## 3. Results

This study explored the stories of eight older adults—Julia, George, Murray, Stacy, Anna, Eliza, David, and Carol (pseudonyms were used to protect privacy while assisting the reader in envisioning participants as humans)—with monitoring technologies. This research revealed that these individuals, regardless of their cognitive impairment status, adopted a pragmatic approach to living with such technologies. Their experiences could be summarized into four key themes: the freedom to age in place, the necessity for active intervention, individual approaches to technology based on personality/cognition, and the distinction between a self-unchanged and situation changed.

### 3.1. Demographic Information

These eight older adult residents living in long-term environments, with five participants recruited from Missouri and three from Arizona, participated in phenomenological interviews, 4 with dementia diagnoses and 4 without (see Table 1). Cognitive functioning, as assessed by the MoCA, also varied across participants and sites, with mean scores reported for descriptive purposes only. Missing data were noted for age and MoCA scores in a small number of cases.

### 3.2. Freedom to Age in Place

Older adults reported that monitoring technologies provided them with a sense of comfort and freedom by enhancing their feelings of safety through supervision. For Julia, she noted that the presence of monitoring technologies made her more likely to stay in the facility where she lives, which primarily offered independent services, giving us the title for this theme:

*“With this technology, I’m more inclined to stay where I’m at. It gave me the freedom to age in place.”*

Several residents commented on how technology could, or could not, influence daily routines, which added to their lived experiences through *additional support*. Anna interpreted the device as an unobtrusive addition to her routine. Her use of the word *support* stood out, exemplifying how the monitoring technology did not interfere with her daily lived experiences, but instead added meaningful monitoring, enabling aging in place. Anna said:

*“I could live out my life where I currently live, uh, with additional support, as needed, and, um, not have to worry about getting used to new surroundings. Um, I guess you, um, might think…. It’s an additional monitor of your well-being. It didn’t change how I thought about myself. I’m not what you would call a really modest person.”*

Likewise, David was almost dismissive when asked about how the presence of technology influenced his daily routines. *I just didn’t think about it at all*, he said with a shrug. When pressed to think about technology and its presence in his aging life, David turned introspective, then responded in light of his changing needs with a progressing neurodegenerative illness.

*“My arms are not strong to get anything out of the way…my legs are not strong for that. Maybe that’s what they’re telling me… they have to push you… until you’re in the homes, I guess they age you in place. That’s what they’re going to use since I aged in place.”*

Initially, David did not seem to express feelings of freedom so much as resignation to a life lived pushed into a care home. But when asked to explain how the technology influenced his ability to age in place, he responded *I can stay in my room as I get sicker*. In the light of his illness, David found value in the technology as a facilitator to age according to his own terms within a facility with nursing care as his needs progressed.

Eliza expressed disappointment that the study funded the monitoring technologies was ending because its presence made her feel better. She felt reassured with the technology present and its absence brought forth the reality that she would miss the feelings of security and wellbeing it enabled. The unobtrusive nature of the technology experience increased the feelings of independence from being monitored, creating a net positive for her lived experience.

*“Well, I was sorry it was going…I was glad it was watching me because, you know I didn’t want to fall anymore. I was already hurting from the falls I did have. I sure didn’t want to fall anymore.”*

Interestingly, among the three residents with the highest degree of dementia, responses to questions about aging in place and comfort levels with technology were more dichotomous and straightforward. However, they still expressed a sense of comfort in staying where they lived and felt at ease knowing they were being monitored. Given her dementia, Carol used an adaptive communication technique to describe a situation in which she had experienced an injury at home and needed to call her daughter for assistance. She intertwined this story with her current feelings about monitoring technologies in an interesting weave between past and future. Through her tale of past need, she was able to relay her desire for assistance to remain free from the uncertainty she experienced living without monitoring technologies. Carol continually denied any discomfort with the current system and derived meaning from her impactful experiences, highlighting a story of the past to illustrate how monitoring could prevent potential harm in the future.

*“I’ll tell you all something. When I was in the, I was walking, wheeling into the bathroom with my walker, and I opened the cabinets and I leaned over. To see what was, if I could find some cotton, and I could- and some Q-tips that I could make a snowman with. One year when Christmas came, my foot got caught in one of the wheels and I couldn’t get out. And I said help! help! And they came in to help me and they couldn’t get me out. So they had to call the [Fire Department]. And they had to call the ambulance, come get me, take me to the hospital and see what was wrong with my leg. And then they got me out of it and, you know, they took me to the hospital, they took me into the emergency room.”*

Though some older adults expressed initial discomfort, this was ameliorated by the experience that freedom comes from peace of mind. Stacy expressed a mix of comfort and discomfort with the monitoring system. When asked what it was like to live with technology, she stated:

*“Don’t [like it], because it has no heart. It’s always looking at you.”*

But after further prompting to explain what no heart meant to her, Stacy shrugged and changed the tone of her voice. She confided that though she was initially uncomfortable with the presence of technology, it did not change her daily routine. In fact, over time, she changed her approach to technology to one of pragmatism. Stacy was able to shift from one of discomfort to seeing the value of technology for aging in place.

*“It’s there for a reason. A good reason. It means you can live at home and without being in danger of falling or anything.”*

Even though the older adults had different initial reactions, ultimately freedom was gained with little expense in the way of burden. Technology permitted a desired life free from intrusion or over-intervention. The lived experience meant that the older adults did not feel they had lost freedom, instead they had gained freedom by allowing monitoring, which meant they could remain in a place of their choosing. Aging could be realized as they envisioned—at home.

### 3.3. The Necessity for Active/Integrated Intervention

For technology to gain meaning in their lives, how it was used was an essential quality used to assign meaning and create the lived experience. When assigning meaning to the lived experience of older adults with monitoring technologies, most expressed a desire for meaningful systems that contributed to safety and security. As older adults aged and recognized the need for further assistance, technology could be a meaningful adjunct to their lives, but judgment was based on pragmatic usefulness. To truly be appreciated, the older adults wanted technologies that were attached to a care system with actual human caretakers.

Murray noted that monitoring technology could be helpful as individuals need further assistance, but only if it was tied to real care. Reflecting on his own journey from an independent professional to becoming dependent upon others due to impaired vision and his use of an assistive walking device, he was pragmatic about the potential advantages of technology.

*“Well, it alerts somebody to come and help you [but] I think I’d have to have a human.”*

The use of the word “human” reveals that monitoring technology lacks soul and purpose in Murray’s life, and it is only the care enabled by the technology that matters. It is the people and carers that bring purpose to Murray. In the event of an emergency, it is the community that responds (be it neighbors or nursing caregivers in a facility), and this is what allows technology to have meaning and be incorporated into Murray’s lived experience as he explained with a story of his home and neighbors:

*“Well. I might give an example. I lived in the country right at the edge of the city limits, and you know we always have storms, or we potentially have storms. A friend of mine, and I bought an electric generator that, if we have an ice storm it breaks all the power lines, any- for any interruption, that generator comes on and replaces [power]. Now that I’m not in my home, I have the satisfaction of knowing if we have an ice storm or a windstorm, uh, my house is probably heated or cool, especially heated in the wintertime. And somebody lives on the farm, checks my house and- and checks my mail, and so on. That’s what I’m thinking of in terms of the technology.”*

Anna, who lived in the same facility as Murray, expressed similar skepticism about technology if it was not supported by a meaningful care system. She expressed disbelief that current technologies could reach the level of system sophistication needed for meaningful integration and alerts to support aging in place. However, she adopted a future-minded perspective, drawing on her experience with existing monitoring technologies to improve future systems.

*“If you have anything [any technology], you have to have, also, the staff trained to respond to the signals that are sent. It’s not just the technology that determines the value, it is the system, and the system is just a part of the bigger system. I- I knew that probably in my lifetime, that, uh, the technology would not reach that level. So, uh, I thought of it more in the future sense of, uh, other people, other than myself.”*

The participant’s descriptions of technologies reveal current gaps in how care is provided and questions if current care structures can support the level of intervention(s) needed as indicated by monitoring technologies. Although he appreciated having monitoring technology in his room, Murray expressed skepticism about whether the long-term care system could adequately respond to alerts. By describing care as fragmented, Murray made clear that current care systems were not streamlined enough to appropriately incorporate additional information provided by technology.

*“They have their work cut out for them. You have a CNA (certified nursing assistant) to help you with bathing and dressing and so on, and you have someone else that delivers your medication… different levels of specification… it seems like the care is pretty chopped up.”*

Likewise, Anna felt the presence of technology alerts would be insufficient because she doubted there would be a coordinated response or enough staff available to assist her when needed. The lived experience meant that Anna felt a distance from the technology, because current care realities made it less than useful.

*“It’s- the nursing staff here can’t- can’t handle an additional challenge of having something like- like a monitoring system of any kind triggering them. [The nurses] require different training, and probably, a [higher] staffing level.”*

Care systems enabled by technology also need to be proactive to truly meet the needs of older adults with potentially rapidly changing health status. James added perspective on the importance of proactive vs. reactive systems. He relayed a story of a recent event which happened after a monitoring sensor was removed, leaving him feeling unprotected. We find that James kept a distance from the technology because he did not find it useful for his health needs.

*“Those things don’t help you before- before you the, the (he gestures at the ground) stupid floor. It’s not that very reassuring when you hit the ground, let me tell you.”*

All the individuals interviewed expressed a desire for remote or integrated solutions that did not require residents to interact with the devices, press buttons, or manage them themselves. The extra burden of managing a device detracted from a meaningful experience and created unnecessary perceived burden. Especially when considering older adults with dementia, technology that requires pushing buttons or interfacing with readouts can be prohibitive. Julia highlighted that there were times when the button may not be accessible, referring to a pendant monitor that required the resident to press it for help. She expressed doubt that a resident with dementia could reliably interact with a device that required ongoing care and maintenance.

Eliza, who had a diagnosis of dementia, had similar reservations framing her value judgment on her experiences with technological shortcomings. The lack of relevant features meant that Eliza could not fully incorporate technology as meaningful to her lived experience.

*“[it required] the nurse… to reset something. I like it in the ceiling… cause it’s up above… and saw when I fell, if I fell.”*

Thus, the how matters more than the what. For these participants, meaning can only be felt when the technology modality and the care system can be activated in proactive ways. Expectations for monitoring usefulness were used to interpret the lived experience and, therefore, determine acceptance or rejection of the technology for meaningful aging.

### 3.4. Individualized Approaches to Technology Based on Temperament, Usefulness, and Worldview

Participants adopted a pragmatic approach to monitoring technology by grounding their reactions in personal temperament, perceived usefulness in daily life, and, for some, a willingness to improve the future. Older adults without cognitive impairment tended to adopt a forward-thinking, altruistic perspective, focusing on improving care systems for future generations. In contrast, older adults with cognitive impairment or dementia evaluated monitoring technology based on past experiences and on its day-to-day influence in their lives.

Murray, who had no dementia, approached the technology with a philanthropic mindset, aiming to improve technology to the benefit of others. Murray valued education in his life and for his family, and the monitoring technology allowed him to express this value. His intact cognition allowed Murray to think in abstract ways that could then be leveraged to provide meaning to living with technology.

*“Um, just like I’ve worked with these nurses (referring to student nurses), and give them a so-called guinea pig, and they can make assessments on me. And I felt the same way about your project. Whatever I can do to help.”*

Julia was also enthusiastic and welcoming when visited for her interview. She was eager to share her perspectives and expressed gratitude for the opportunity to participate in the study on monitoring technologies. She focused her responses on how her involvement could help improve care through improved care systems to enable aging in place. Julia’s identity as a valued member of society contributing to future good was reinforced by using the monitoring technology and providing feedback.

*“It’s a progressive part of life, I can’t imagine having to go to two-hour checks. It’s important to help support new ideas and further education. Aging in place allows me to become better in one place, get therapy, yet I can stay until it’s too unsafe and then get the care I need. I can make this place my home.”*

In comparison, older adults with cognitive impairment used simpler language and shorter responses grounded in their daily experiences, yet they were still able to express how technology had influenced their lives. Carol presented one of the most challenging interviews, often responding with, I don’t know, even when questions were simplified. However, with gentle persistence and time and space to expand her thoughts, she began to share her experiences through storytelling, weaving between past events and current realities. Carol’s focus on simple language and vacillating between past and future indicates her lived experience was grounded in daily experiences due to her cognitive impairments. By giving time and space for Carol to tell her story, and by being present in the moment dwelling in Carol’s world, we were able to interpret and bring meaning to Carol’s tale.

*“If it rains, then a search, then a caretaker would come in, one of the people that worked there [would come] in and find me. It changed, they say, what happened? They say, then they take me to the hospital, and they take, X-ray my leg, or my arm, or my, whatever was hurt, and then they tell me, you got a sprain, or something is wrong with you.”*

When asked how technology affected her self-perception, Stacy denied any changes in her routine but also expressed discomfort in technology watching her. Stacy eventually found comfort and could balance being monitored continuously by acknowledging its usefulness in her daily life.

*“I want one that tells you… more information. It makes me feel comfortable. They know it, ok, so the alert, knowing that it alerts folks makes me comfortable.”*

Though the lived experience with technology was interpreted through individual life experiences, personalities, cognitive states, and worldviews, the value calculation older adults made to determine technology value was remarkably similar. As health changes over time, the participants assigned meaning through language, objects and even daily interactions using personal approaches grounded in identity and cognitive abilities. The presence of technology had the potential to alter or change the definition of self for each aging adult as they transitioned and incorporated technology into their lives. We found the language used by the older adults emphasized value and usefulness from their own unique perspectives, ultimately converging on the idea that technology could be useful to their sense of self, enabling an independent life that they value.

### 3.5. Situation Changed/Self Unchanged

Throughout their lived experiences with monitoring technologies, all participants emphasized that the technology had little to no effect on their sense of self or identity. Instead, most participants noted that monitoring technology was a welcome and sometimes necessary addition to their changing life situations as they grew older and needed more assistance.

Julia and David approached technology with a strong sense of self, and the technology which was enabled by the remote modality. Julia mentioned that she often forgot the technology was even present, saying, *I had to remind myself it was there*. David emphasized that the technology embedded in his room did not influence his daily life. *I didn’t notice any difference at all*, he said. When asked how the technology influenced his feelings about himself, he replied, *It didn’t matter*.

Murray, however, approached technology with a sense of resignation, but then altered his perspective to fit the technology within his image of himself as an older adult needing more assistance over time, rather than altering his life and self as an adult dependent upon the technology. He had maintained the same outlook and desire for independence as he aged, but he recognized the need for help as his mobility declined and his eyesight diminished; technology had become another means of assistance.

*“(You) want to be as independent as you can be here, do as much for yourself as you can… but I don’t have a choice.”*

For older adults with dementia, their sense of self was maintained. Though less explicit, their thoughts about changed situations influenced how they interpreted their lived experiences with monitoring technologies. Eliza had initially found it strange to be monitored, but she later felt grateful for the oversight. The technology made her feel safer as she had still been experiencing the physical effects of previous falls and recognized that she was at risk of future harm. Pragmatism and recognition of a changed situation eased her initial discomfort, resulting in a consistent sense of self and an acknowledgement of the need for assistance to live a safer and therefore fuller life as she aged in place.

*“It [the technology] would be good, I’m probably going to stay here forever.”*

Stacy initially felt uncomfortable with the technology; however, she expressed a desire to have it installed because of her feelings about impending mortality. I wanted to die, she said, because of flexible life, where I’m cooked, she added, motioning to her wheelchair. In her field notes, the PI recorded her initial concerns about suicidal ideation. However, with further gentle prompting, Stacy clarified that she felt saddened by her reduced physical condition, which required her to use a wheelchair, and by the limited opportunities that came with her limited mobility. Technology did not change or cause her feelings of helplessness; rather, her emotions were commensurate with her changing life situations. Ultimately, the presence of technology:

*“Makes me feel comfortable… whenever I’ve [injured], they know it”*

Aging in place was enabled through technology, which helped older adults maintain their feelings of autonomy and independence. Enabling independence, even in less independent situations such as aging bodies and minds, helps to maintain a core identity that transcends situation, unaltered by the presence of technology in their lives.

## 4. Discussion

While monitoring technologies hold promises to improve the lives of older adults by supporting assisted independence, it is essential to consider how they perceive their daily lives with technology. Understanding their perspectives is crucial for ensuring that these interventions are effective and truly desired. The lived experiences of older adults with monitoring technology have been studied, but most research focuses on caregivers’ perspectives or on older adults with intact cognitive function [11,12,13]. Research primarily focuses on usability, rather than on the meaning or formation of identity. Notably, even though older adults with dementia are often the primary target for these technological interventions, they are frequently excluded from research. This exclusion occurs due to the progressive decline in cognitive function, which includes impairments in complex and expressive language which are essential skills for providing deep descriptions and ensuring informed participation in qualitative research [19].

In this interpretive phenomenological study examining the lived experiences of older adults with and without dementia, the inclusion and comparison of both groups significantly enhanced our understanding of how to create person-centered care when implementing monitoring technologies [19]. We found that even when the answers were mainly binary or if older adults with dementia seemed to ramble in their replies, there was often a core of depth and understanding that could be identified and extracted. This was especially true when the interviewer allowed adequate time and space for expression. Older adults with dementia use compensatory communication mechanisms such as storytelling [20]. When they receive active listening and gentle redirection, they often eventually answer questions. Higher-order concepts such as aging, self-identity, and aging in place, which involve looking toward the future, can be better understood when double hermeneutics are applied. This approach helps interpret meaning within context during a communication exchange that is developed patiently over time between the participant and the interviewer [19,21].

The older adults in our study used pragmatic approaches to create meaning in their lived experiences with monitoring technologies. Although self-identity remained intact, changes in life situations experienced with aging minds and bodies were also considered when accepting monitoring technologies into their lives. Older adults conceptualize independence beyond traditional measures, such as completion of activities of daily living, and instead view independence as a mix of form and function. Form consists of observable behaviors while function consists of roles and purpose that allow the older adult to maintain valued identity [22]. Our study indicates this is true as older adults were able to conceptualize role function as important indicators of self-identity. Their lived experiences with technology incorporated an understanding and value judgment of how technology can assist with maintaining form and function as the person ages. Aging in place was tied to more than physical location; it was tied to a location and care system that helped older adults achieve form and function that aligned with their ideas of independence [22,23].

Similarly, we noted that older adults’ perception of technology was influenced by place and technology modality. Older adults placed value judgments on the usefulness of technology based on care systems where they lived and how they were expected to interact with care systems. Perceived needs for maintaining independence change over time and living situation [22]. This was true in our sample as well, as older adults used personal experiences, needs and care preferences to assign meaning to the lived experience with technology, including where care was received. Independence was partially determined by “form” of circumstances, including place (i.e., long-term care) as well as function, including interactions with the technology and care systems [22]. Our sample included older adults who had already made a decision to receive care in a formal manner in a congregate setting and therefore this reality was incorporated into their lived experiences with the technology. Specifically, the participants interpreted usefulness and assigned value based on how the technology was incorporated into existing care systems where they lived. Likewise, participants expressed a clear desire for remote technologies which reduced burden and remained in the background of the lived experience.

The Technology Acceptance Model (TAM) predicts an individual’s likelihood of accepting and adopting new technology based on perceived ease of use and perceived usefulness [24]. We found that older adults used these features when interpreting the meaning of technologies. For example, participants preferred remote technologies over wearable technologies because of their perceived ease of use, as these systems could be installed and did not require maintenance from older adults. Older adults found monitoring technology useful only when connected to meaningful, human-based care response systems. However, a drawback of the TAM is that it presumes people make decisions solely based on technology’s usability and neglects the importance of the self and how technology can influence or even threaten one’s self-identity. In our study, we found that older adults considered not only the usability aspects of monitoring technology but also how it influenced their life course, specifically in enabling aging in place. Understanding how older adults perceive the concept of aging in place reveals their feelings and attitudes toward self-identity as they face declining physical and cognitive capabilities. It also highlights how monitoring technology can influence this experience, both currently and in the future.

Carter and Grover (2015) conceptualize information technology (IT) as a means of forming an identity that influences human behavior, which they term IT identity [25]. Identity can be conceptualized as an individual’s understanding of self, using symbolic interactionism to assign meaning to the roles, relationships, and objects that define who they are, in other words, the question of “Who am I?” [26,27]. IT can be considered an object that individuals interact with as they shape their sense of self. This relationship yields an IT identity that can be fully understood only by querying individuals about the meaning of IT in their self-conception [25]. As various forms of IT pervade society, understanding how technology and information shape how people “express, maintain, and expand their self-concepts” is an integral and inseparable aspect of life. This study acknowledges the reality of monitoring technologies and intentionally includes end-users to understand its influence on identity and self, specifically regarding the dimension of aging.

IT identity can be further influenced by Human–Computer interaction (HCI), the way older adults (humans) choose to use and interact with computers (technology). While IT identity focuses on how technology becomes part of one’s self-concept, HCI helps explain why this integration succeeds or fails, particularly through interaction burden, perceived control, transparency, privacy, and the embedding of technology within socio-technical care systems [28]. Participants’ pragmatic evaluations of monitoring technology appear to be grounded not only in perceived usefulness, but in their lived interaction experience with the system (e.g., passive vs. active interaction, need to press buttons, reliability of human response) [29]. These findings suggest that IT identity formation may depend on whether the interaction design supports older adults’ autonomy, dignity, and lived routines [30]. When monitoring technologies are perceived as intrusive, opaque, or disconnected from meaningful human response, they are unlikely to be internalized as part of one’s identity, regardless of their technical capabilities. Older adult wellbeing is strongly associated with the type of care network (e.g., formal paid caregivers, none, or informal carers) from which they receive support, mainly through social connectedness and loneliness [31]. If technology is not perceived as a valuable network which reduces loneliness and increases social connectedness, it may be deemed unacceptable to the older adult and therefore not incorporated into identity or add value to the lived experience. Integrating HCI principles into the design and implementation of monitoring systems may therefore be essential for supporting aging in place in a manner that aligns with older adults’ self-concepts.

Our findings suggest that both individuals with and without dementia understand how technology can be incorporated into daily living to assist with aging; however, they still fall short of incorporating it into their sense of self. The pragmatic approach our participants used to understand the role of technology in their lives indicates that IT could become part of an internalized IT identity if usability and preference concerns are addressed when implementing monitoring technologies through HCI. When personal perspectives are not sought or respected, barriers to IT identity development arise, threatening usability [30]. For example, our participants appreciated that technology could monitor their health. Still, they felt it was useless unless linked to real-world interventions, which could assist older adults in the event of a fall. Monitoring technology is sometimes viewed as a threat or creates discomfort for users due to perceived privacy risks or its limited usefulness for aging in place. Without personal integration into a meaningful life course, the technology is not welcomed.

Understanding how older adults identify with and assign value to technology provides a guidepost for future technology interventions intended to monitor older adults. Successful health implementation requires knowledge of more than just the technical and clinical, but also a knowledge of desires, perspectives and concerns of all end users. According to Rachmad’s Motivation in Health Theory, health motivation is “a living process shaped by emotion, cognition, culture and identity”, therefore activating an older-adults motivation to participate and maintain health is based in understanding their lived ecosystem [32]. As older adults age, focus turns to quality of existence measures, such as psychological and emotional wellbeing, instead of just performance. Control, autonomy, inclusion, and respect are valued [32]. For a health intervention to succeed, the change must be grounded in human experiences and knowing how people act in presence of others [33]. Therefore, technology implementation meant to target and improve older adult health and wellbeing should include understanding of the older adults themselves and their care environments, which can only be elucidated by seeking older adult end-user perspectives in a process of co-creation. Technology interventions have the potential to mediate how older adults interact with their self-identity and environment but only if they are realized in human-centered and empathetic ways. The assumption that technology can effectively ease the “burden” of caregiving as the population of older adults grows is often cited as a justification for implementing these systems. However, there is often little consideration given to the perspectives and desires of the older adult users themselves [31,34]. In fact, most technology research tends to focus on older adults’ privacy and autonomy instead of their feelings of safety and self-identity. This perspective approaches aging as a problem to be solved, reflecting an ageist approach. It prioritizes managerial objectives such as control and organizational quality improvement, over individual health and personal choice [34,35]. For older adults with cognitive impairment, technology is often viewed as a standard of care and their denial to participate with monitoring technology can be seen as “resistance”, a hurdle to overcome [36]. Our study found that older adults with dementia were not only able to convey their acceptance or hesitancy towards technology but also to articulate the meaning and impact of technology on their lives, rooted in their personal experiences, when provided with adequate time and space.

By moving away from assumptions about benefits and an interventionist mindset, we approached and conceptualized aging and technology as being “co-constituted.” Per Peine and Neven (2021), it is essential to examine technology and aging together to “bring each other into existence” [37]. This approach aims to bridge insights from aging research with findings from science and technology research. Through the co-constitution of aging and technology (CAT) model, we acknowledge that technology must be studied in both the design world and the everyday life context, as they cannot be understood separately. Our older adult participants demonstrated how they used both past and current life experiences with technologies to evaluate the current and future relevance or usefulness of technologies as they navigate life and aging. Technology exists within the “life-world” of the older adult, and only by exploring and eliciting these life-worlds can we understand the value and ethical application of these technologies to refine and improve their design [34,37].

### Limitations

We must acknowledge several limitations that could influence the transferability and validity of our findings. First, this study involved a small, purposively chosen group of older adults. However, given our focus on exploring depth and meaning through qualitative interviews regarding a specific topic or experience, a small sample size is to be expected, and purposive sampling is preferred in this context. Additionally, we acknowledge that our findings may not be generalizable to other settings. However, given the focus on understanding meaning and self-identity in the context of monitoring technology, we focused on transferability over generalizability. We believe this study can provide valuable insights transferable to similar contexts such as interventions and applications involving older adults and technology in aging-in-place environments.

## 5. Conclusions

Older adults, both those diagnosed with dementia and those without, often serve as the target demographic for monitoring technologies. However, their perspectives are frequently overlooked in the discourse surrounding these innovations. This study significantly contributes to the understanding of effective implementation strategies for monitoring technologies and explores the relationship between technology and older adults’ perceptions of their identity.

The findings of this research offer valuable insights for individuals and organizations aiming to successfully integrate health monitoring and prevention technologies, thus helping older adults to maintain their independence and age in place effectively. Even as older adults experience cognitive decline, their perspectives and lived experiences are invaluable and need to be intentionally sought out.

Without thoughtful design and the inclusion of older adult end-users, technology interventions risk undermining important values for older adults, such as autonomy and independence, which could paradoxically hinder their ability to age in place. It is crucial that technology interventions are co-created with older adults, including those with dementia, to design programs that are truly meaningful and relevant.

## Figures and Tables

**Table 1:** Demographics.

Characteristic	Overall (N = 8)	Site	
		Missouri (n = 5)	Arizona (n = 3)
**Gender**			
Female	5	2	3
Male	3	3	0
**Age range (years)**			
65–69	2	0	2
75–79	2	1	1
80–84	2	2	0
≥85	1	1	0
(Missing)	1	1	0
**Dementia**	4	2	2
**MoCA score**	16.20 (5.02)	20.00 (4.24)	13.67 (4.16)
(Missing)	3	3	0

Note: Values are presented as the number of participants unless otherwise indicated. MoCA scores are reported as mean (standard deviation) for descriptive purposes only.

## Data Availability

The datasets presented in this article are not readily available because of privacy concerns with primary interview transcripts. Requests to access the datasets should be directed to Alisha H. Johnson, ahjfbk@missouri.edu.

## Abbreviations

The following abbreviations are used in this manuscript:

IoT	Internet of Things
IPA	Interpretive Phenomenological Analysis
PI	Principal Investigator
Co-PI	Co-Principal Investigator
MoCA	Montreal Cognitive Assessment
TAM	Technology Acceptance Model
IT	Information Technology
HCI	Human–Computer interface
CAT	Co-institution of aging and technology
